# Comparative micro-epidemiology of pathogenic avian influenza virus outbreaks in a wild bird population

**DOI:** 10.1098/rstb.2018.0259

**Published:** 2019-05-06

**Authors:** Sarah C. Hill, Rowena Hansen, Samantha Watson, Vivien Coward, Christine Russell, Jayne Cooper, Steve Essen, Holly Everest, Kris V. Parag, Steven Fiddaman, Scott Reid, Nicola Lewis, Sharon M. Brookes, Adrian L. Smith, Ben Sheldon, Christopher M. Perrins, Ian H. Brown, Oliver G. Pybus

**Affiliations:** 1 Department of Zoology, Edward Grey Institute, University of Oxford, Oxford, UK; 2 Department of Zoology, Edward Grey Institute, University of Oxford, Oxford, UK; 3 Department of Virology, Animal and Plant Health Agency – Weybridge, Woodham Lane, New Haw, Addlestone, Surrey KT15 3NB, UK; 4 The Royal Veterinary College, Royal College Street, London, UK

**Keywords:** avian influenza virus, wild birds, H5NX, epidemiology, serology, genetics

## Abstract

Understanding the epidemiological dynamics of highly pathogenic avian influenza virus (HPAIV) in wild birds is crucial for guiding effective surveillance and control measures. The spread of H5 HPAIV has been well characterized over large geographical and temporal scales. However, information about the detailed dynamics and demographics of individual outbreaks in wild birds is rare and important epidemiological parameters remain unknown. We present data from a wild population of long-lived birds (mute swans; *Cygnus olor*) that has experienced three outbreaks of related H5 HPAIVs in the past decade, specifically, H5N1 (2007), H5N8 (2016) and H5N6 (2017). Detailed demographic data were available and intense sampling was conducted before and after the outbreaks; hence the population is unusually suitable for exploring the natural epidemiology, evolution and ecology of HPAIV in wild birds. We show that key epidemiological features remain remarkably consistent across multiple outbreaks, including the timing of virus incursion and outbreak duration, and the presence of a strong age-structure in morbidity that likely arises from an equivalent age-structure in immunological responses. The predictability of these features across a series of outbreaks in a complex natural population is striking and contributes to our understanding of HPAIV in wild birds.

This article is part of the theme issue ‘Modelling infectious disease outbreaks in humans, animals and plants: approaches and important themes’. This issue is linked with the subsequent theme issue ‘Modelling infectious disease outbreaks in humans, animals and plants: epidemic forecasting and control’.

## 1. Introduction

Highly pathogenic avian influenza viruses (HPAIVs) pose a continued and serious threat to human and animal health. Additionally, the spread of HPAIV to farmed poultry can result in great economic damage, through reduced production, trade restrictions and the direct costs of emergency control measures such as livestock culling [1]. While wild waterbirds are the main host reservoir for low pathogenicity avian influenza viruses (LPAIVs), most highly pathogenic strains have in the past been detected primarily within poultry [2] or in humans, where they occasionally cause serious disease [3]. However, one widespread and diverse lineage of HPAIV deviates from this paradigm: viruses belonging to clade 2.3.4.4 of avian influenza H5 subtype and that descend from the GsGd lineage (A/goose/Guangdong/1/1996) appear to transmit efficiently among wild birds [4]. The propensity of this virus towards circulation in wild birds has been associated with virus diversification via genome reassortment and with its unprecedented global spread. Strains of the GsGd lineage have been detected across North America, Africa, Europe and Asia [4].

In the context of the emergence and persistence of H5 HPAIV, understanding the epidemiological dynamics of avian influenza in wild birds is critical to the development of effective surveillance and control measures that aim to safeguard the health of livestock and human populations. Data on HPAIV in wild birds at each outbreak location are typically limited to the date of confirmed virus detection, the number of dead birds, and the host species involved. Compilations of such reports are used to study the geographical and temporal distributions of detected HPAIV cases in wild birds over continental or regional scales [5–8]. By contrast, detailed population-level analyses of the epidemiology and transmission of HPAIV for individual outbreaks are rare. Almost no data are available on the impact of sequential outbreaks or virus incursions into the same wild bird population, despite the existence of at least one site where HPAIV has been detected on five different occasions [9]. The scarcity of individual-level information for wild bird populations affected by HPAIV means that key epidemiological parameters that could be used to refine surveillance and control measures remain unknown.

Understanding the immunological responses to HPAIV in wild birds is necessary to understand and predict the risk of the virus becoming enzootic in wild bird populations. In Europe alone, serological studies suggest that up to a third of wild birds from some species may have been infected by HPAIV [8]. However, in most wild populations, it is difficult to capture the same bird multiple times over long time periods, making it hard to evaluate the duration of avian influenza virus (AIV)-specific immune responses, or the degree to which initial exposure might help to protect against subsequent infection. Experimental challenge studies in the laboratory have shown that primary infection of ducks with some H5 HPAIVs, or with certain LPAIVs, can prevent virus shedding upon subsequent challenge with H5 HPAIV (e.g. [10,11]). However, such studies are often limited by the experimental requirement of a short time period between primary and secondary infection, making it difficult to assess the protective effect of previous infections in wild bird populations, where primary infection may occur years before secondary exposure. Quantifying the duration over which wild birds retain antibodies to HPAIV and LPAIV, how these responses are distributed within a population, and how they influence susceptibility to subsequent exposure to H5 HPAIV, are all important for understanding the biological basis of the infection patterns that we observe worldwide.

Here, we report the detailed ‘micro-epidemiology’ of recurrent H5 HPAIV outbreaks, which occurred in a single wild bird population while that population was under longitudinal sampling and observation. This unusual scenario allowed us to quantify serological responses within the affected population before and after an HPAIV epidemic. Our study population comprises a large population of mute swans (*Cygnus olor*) that have been naturally infected by three different HPAIV viruses belonging to two genetic clades (2.2 and 2.3.4.4) over the last decade; H5N1 in 2007/08 [12], H5N8 in 2016/17 and H5N6 in 2017/18. Importantly, these birds have been the subject of ornithological study for more than 60 years, and therefore detailed demographic information is available for the population and the individual birds within it. The population is therefore particularly suitable for exploring the natural epidemiology, evolution and ecology of HPAIV in wild birds at a high degree of precision and certainty. We show that key epidemiological features are consistent and predictable across multiple outbreaks in a complex natural population and we discuss the implications of these findings for HPAIV transmission in wild birds.

## 2. Material and methods

### (a) Field site, population and sampling

The Fleet Lagoon (Dorset, United Kingdom, 50.6537° N, 2.6028° W) is home to a large population of wild mute swans (*Cygnus olor*), centred on the Abbotsbury Swannery. The population size changes seasonally and between years, but typically ranges between 600 and 1000 birds. Swans hatched at the site are tagged with unique ID markers approximately 24 h after hatching that are replaced with adult rings at approximately four months of age. Relatively few birds that hatch locally move away from the Fleet [13]. Every 2 years, all swans present on the Fleet Lagoon are caught, ringed, weighed, and their year of hatching and/or sex recorded, where possible. As a consequence, detailed data about the date of hatching and sex are known for most swans on the Fleet Lagoon. Individual birds can be identified by unique IDs that they carry on two different leg rings (one metal ring, supplied by the British Trust for Ornithology, and one Darvic ring, which allows long-range visual identification). Birds thought to have been in the population at the time of each outbreak were estimated from census data, according to details in the electronic supplementary material. Note that in this paper, we use the term ‘juveniles’ to refer to birds that are less than 1 year old, and ‘adults’ to refer to birds that are older.

A total of 519 blood samples were collected from 404 swans on the Fleet Lagoon during June 2017, July 2017, November 2017, January 2018 and June 2018 (UK Home Office licence PPL P516CDFB6). A cloacal swab and an oropharyngeal swab were also taken from every bird that was blood sampled.

### (b) Serological testing

Sera were tested for the presence of antibodies directed at the AIV nucleoprotein (NP) using the AIV IDEXX Influenza A Ab ELISA, according to the manufacturer's instructions. Haemagglutination inhibition (HI) assays were conducted for each sample that tested positive by NP-ELISA using two inactivated antigens, LPAIV H5N3 (A/Teal/England/7394-2805/06) and HPAIV H5N8 (A/Turkey/England/052131/16). HI assays were conducted according to standard methods [14] (further details are provided in the electronic supplementary material).

### (c) Epidemiological surveillance during HPAIV outbreaks

Dead swans found at the Swannery site are reported by staff to the Animal and Plant Health Agency (UK) (APHA) and may be subjected to post-mortem and testing for notifiable diseases. Following detection of HPAIV H5 at the site in December 2007, December 2016 and December 2017, oropharyngeal and cloacal swabs were collected from all dead birds of any species, where possible, and processed at APHA (further details of site surveillance across outbreak years are provided in the electronic supplementary material).

Not all birds that died during the H5N8 outbreak could be tested for AIV. The epidemiological analyses for the H5N8 outbreak undertaken here (see below) therefore assume that untested dead birds found during the H5N8 outbreak were HPAIV-positive (see electronic supplementary material, figure S1 for details of which birds were tested). The typical age-adjusted mortality rate of birds in the same weeks during non-epidemic years is dramatically lower (approx. 12 recorded deaths per 1000 birds; data averaged between 2009 and 2015) than the mortality rate observed during the peak of the H5N8 epidemic (approx. 143 deaths per 1000 birds; based on contemporary population size). Thus, we can reasonably assume that almost all of the untested birds at the time of the H5N8 outbreak died of HPAIV.

### (d) Viral RNA detection and sequencing

Individual cloacal and oropharyngeal swabs were tested for the presence of AIV at APHA. RNA was extracted using QIAamp Viral RNA mini kits and samples were tested for the presence of AIV RNA using previously published reverse transcriptase quantitative PCR (RT-qPCR) protocols [15–17] (see electronic supplementary material for details).

H5N8-positive samples from January 2017 were reverse transcribed, amplified using a multiplex PCR method and sequenced using the Oxford Nanopore Technologies MinION device, following an adaptation of previous methods [18]. H5N6-positive samples from January 2018 were sequenced on an Illumina MiSeq using a non-specific metagenomic approach (see electronic supplementary material for more details of sequencing approaches).

### (e) Phylogenetic analysis

A dataset of Eurasian H5 strains with collection dates in or after 2013 was downloaded from GISAID (www.gisaid.org) and used for phylogenetic reconstruction (see electronic supplementary material for dataset details). Alignments for each segment were completed using the version of MUSCLE implemented in Geneious 8.1.7.

Molecular clock phylogenies were estimated for the AIV HA gene using the Bayesian Markov chain Monte Carlo (MCMC) approach implemented in BEAST [19–21]. Appropriate temporal signal for molecular clock analysis was confirmed using TempEst [22]. Two independent MCMC runs of 150 000 000 steps were computed under a strict molecular clock model, an SRD06 nucleotide substitution model, and a constant population size coalescent prior. Trees were sampled every 20 000 steps, with the first 10% discarded as burn-in. Convergence of the MCMC runs were checked using Tracer [23] and maximum clade credibility trees were computed using TreeAnnotator [24].

For the other seven segments, a preliminary phylogenetic tree was estimated using neighbour-joining, with Jukes–Cantor genetic distances. If several genetically distinct large clades were observed (typically representing reassorted internal genes), then the clade that contained the sequences from this study was extracted. These extracted sequences were then used to estimate maximum-likelihood (ML) phylogenies using PhyML [25], including 100 ML bootstrap replicates to evaluate statistical support. An appropriate substitution model for each ML phylogeny was chosen using the BIC approach implemented in jModelTest [26].

### (f) Epidemiological analyses

Basic reproduction numbers (*R*_0_) for the H5N8 and H5N6 outbreaks were estimated using the R package R0 [27]. *R*_0_ was not estimated for the H5N1 outbreak because very few cases were observed during that outbreak. Numbers of observed swan carcasses were used as a proxy for HPAI case counts. A distribution was specified for the epidemic generation time, obtained from laboratory studies that observed the time between experimental inoculation of geese or ducks with H5N8 and the time to subsequent infection of contact birds [28–32].

The majority of deaths occurred in juvenile birds (less than 1 year old). We tested for possible effects of the last known weight or exact age on the probability of death of juvenile birds. Exact hatch dates are known for almost all birds born into the population, and all juveniles are weighed in September or October of their hatch year. For all ringed juveniles, exact age (in days) was calculated for the day on which they were weighed, and a generalized linear model was fitted to calculate the expected weight of a bird of that age and sex. The difference between the weight of the bird at ringing and its expected weight was calculated. A generalized linear model was constructed to test whether the probability of a bird dying was affected by its age at outbreak start and/or how relatively underweight or overweight the bird was at last weighing.

The case fatality rate (the proportion of individuals that died among all individuals that were infected) was crudely estimated for juvenile birds in both outbreaks. Estimates were not calculated for adult birds because the protective effect of primary exposure to H5N8 upon subsequent exposure with H5N6 would prevent direct comparisons of viral virulence. The number of juvenile birds that had been infected was estimated by summing the number of birds that died during the outbreak and the number of birds that were serologically positive for AIV by HI assay (for H5N8) or ELISA (for H5N6) after the outbreak. As no blood samples were collected prior to the H5N8 outbreak, we assume that juvenile birds were seronegative for H5N8 prior to the outbreak and that the presence of an H5N8 response therefore represents seroconversion following infection. Using NP-ELISA seropositivity as a proxy for seroconversion due to HPAI H5N6 infection appears appropriate because almost all juvenile birds were found to be seronegative by this method before the outbreak. However, we stress that these assumptions make our estimates of the case fatality rate relatively crude. Distributions of the mortality rate among infected individuals were estimated in order to accommodate the effects of sampling and to allow for some uncertainty in the exact number of juvenile birds present in the population in December of each year (electronic supplementary material, figure S2).

## 3. Results

### (a) Comparative epidemiology of the H5N8 2016/17 and H5N6 2017/18 outbreaks

**Figure 1.**
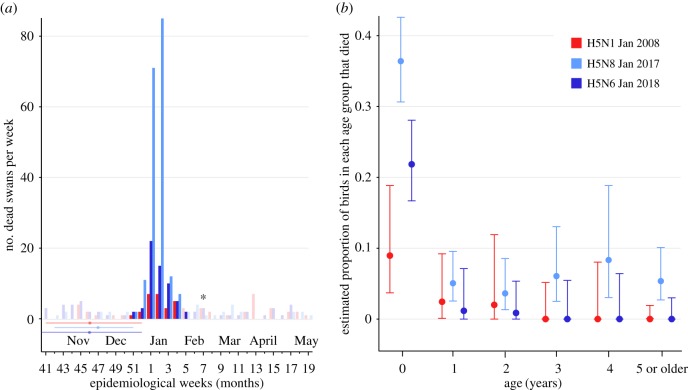
Mortality among swans on the Fleet Lagoon. (*a*) Mortality among swans greater than approximately four months old on the Fleet Lagoon during H5N1 (2007/08), H5N8 (2016/17) and H5N6 (2017/18). Brightly coloured bars indicate the number of dead swans observed during HPAIV ‘outbreak periods’ on the Fleet Lagoon (period between the first and last confirmed positive cases in swans at the site for each HPAIV subtype). Brightly coloured bars include all mortality observed during the outbreak period, regardless of whether the carcasses were tested for AIV or AIV positivity. Pale-coloured bars indicate the mortality observed among birds on the Fleet Lagoon in periods when HPAIV was not detected, and therefore indicate the typical level of mortality observed among swans on the Fleet Lagoon. While the last HPAIV H5N1-positive swan was found in the vicinity of the Fleet Lagoon during week 4 of 2008, a Canada goose (*Branta canadensis*) was found positive for HPAIV less than 1 km from the Fleet Lagoon during week 7 of 2008 and is marked with an asterisk. Dots and horizontal lines indicate the median and 95% HPD interval dates for the MRCA of the Fleet Lagoon outbreak clade, as estimated using phylodynamic methods. (*b*) Estimated proportion of birds of each age group that died of HPAIV infection during each outbreak, based on birds believed to be alive in the population at the time each outbreak started. Colours indicate the respective outbreaks. Adjusted Wald 95% confidence intervals are given.

The three outbreaks of H5 HPAIV at the Fleet Lagoon were strikingly similar in their duration and timing within the calendar year (figure 1*a*). The first cases of each outbreak were detected on 27 December 2007 for H5N1, 23 December 2016 for H5N8 and 31 December 2017 for H5N6. The time between the first and last confirmed positive cases in swans at the site (hereafter referred to as the outbreak period) was 33 days for H5N1, 32 days for H5N8 and 31 days for H5N6.

During each of the outbreaks, several swans were observed to be compulsively spinning on the water. This symptom has been observed only rarely at the site outside of the HPAIV outbreaks. Multiple birds, including one bird that was found to be positive for H5N6, were observed to be lethargic or to have very poor coordination. In the months following all three outbreaks, unusually high numbers of swans with severe torticollis (abnormally twisted necks) were observed.

Crude mortality in the swan population was three times higher for the H5N8 2016/17 outbreak than for the H5N6 2017/18 outbreak (figure 1*a*). For the H5N6 epidemic, 61 swans died during the outbreak period, of which 51 were confirmed by RT-qPCR to be AIV-positive and six confirmed to be AIV-negative. The four remaining birds were not tested, typically because carcasses were incomplete (electronic supplementary material, figure S1). For the H5N8 epidemic, 182 swans died during the outbreak period, of which 18 were confirmed to be AIV-positive and nine AIV-negative. Most of the remaining birds could not be tested because, owing to the scale of the outbreak, it became necessary to dispose of bird carcasses before testing could be conducted. The lower ratio of AIV-positive-to-negative birds in the H5N8 outbreak compared with the H5N6 outbreak is likely because AIV testing took place only at the start and end of the former (electronic supplementary material, figure S1); if testing had occurred during the peak of mortality then a higher proportion of total deaths would have been attributable to AIV. Age-adjusted mortality per 1000 birds during the H5N8 outbreak (=143) was more than double that estimated for the H5N6 outbreak (=65), and more than four times that of H5N1 (=32).

The basic reproductive number (*R*_0_) was estimated for the H5N8 and H5N6 epidemics from time series of the number of recovered carcasses at the site and an estimated epidemic generation time distribution; the latter was obtained from published experimental data for H5N8 viruses. The best-fitting distribution for this parameter was a Weibull distribution with a mean generation time of 2.9 days and a standard deviation of 1. The estimated *R*_0_ values of the two epidemics were similar, although the estimate for the H5N6 2017/18 outbreak (*R*_0_ = 2.69; 95% confidence interval = 1.40–5.5) was considerably more uncertain than that of the H5N8 2016/17 outbreak (*R*_0_ = 2.25; 95% confidence interval = 1.92–2.68). The greater uncertainty in the estimate of *R*_0_ for H5N6 2017/18 likely results from the smaller number of cases observed in this outbreak.

### (b) Molecular clock phylogenetic analysis

**Figure 2.**
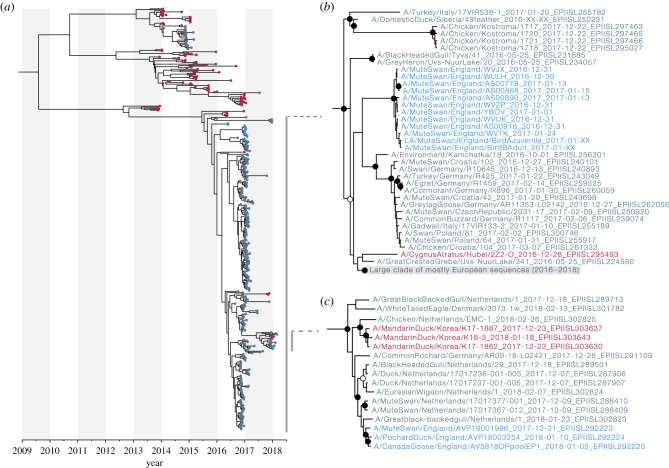
Bayesian phylogenetic trees of HA sequences. (*a*) Bayesian time-scaled phylogenetic tree of the HA segment of 421 Eurasian H5 HPAIVs. Colours at tips indicate the location of sampling (Asia: red, Europe (including Russia): dark blue, Fleet Lagoon: bright blue). (*b*) Expansion of the well-supported clade that contains the H5N8 viruses sampled on the Fleet Lagoon (bright blue). The location of this clade within the larger phylogeny is indicated by the linked vertical grey line in (*a*). Nodes with posterior support values greater than 0.5 or greater than 0.75 are marked with white and black circles, respectively. (*c*) As for (*b*), but showing the phylogenetic position of the H5N6 viruses from the Fleet Lagoon.

Complete or partial virus genome sequences were generated for 12 samples collected during the H5N8 2016/17 outbreak, and for three samples collected during the H5N6 2017/18 outbreak. Preliminary ML phylogenetic trees estimated for each segment showed that, for H5N8, all samples from the Fleet Lagoon formed a single, well-supported clade, consistent with a single introduction to the site. For H5N6, all sequences from the Fleet Lagoon clustered together, but monophyly of this grouping was less robust. Molecular clock HA phylogenies were subsequently estimated (figure 2) in order to estimate the date of introduction of each outbreak lineage into the birds on the Fleet Lagoon. For each outbreak, marginal posterior estimates of the date of the most recent common ancestor (MRCA) of the outbreak clade were obtained from the HA alignment. The median estimated date of the MRCA of the H5N8 outbreak clade was 25 November 2016 (95% highest posterior density credible interval (HPD) = 22 October–21 December 2016). For H5N6, the median date of the MRCA of the outbreak clade was 17 November 2017 (95% HPD interval = 1 October–26 December 2017). These dates are strikingly similar to each other, and to the dates previously reported for the H5N1 outbreak on the Fleet Lagoon (median date = third week of November 2007, 95% HPD interval = middle of October to end of December 2007 [12]). In all three outbreaks, the median date of the MRCA precedes the first observed case and the increase in mortality on the Fleet Lagoon by approximately one month (figure 1*a*).

Based on the estimated molecular clock phylogeny of the HA segment, the H5N8 viruses from the Fleet Lagoon fall within a well-supported clade with viruses that are primarily from Europe (figure 2*a*,*b*). However, branch order within this clade is poorly resolved owing to the high genetic similarity of the outbreak sequences. The divergence date of the H5N8 outbreak clade with the most closely related non-outbreak viruses is estimated to be nearly 1 year prior to the MRCA of the H5N8 outbreak on the Fleet Lagoon. We therefore cannot identify a likely source location for the H5N8 virus that was introduced to the Fleet Lagoon. By contrast, the H5N6 outbreak sequences are placed within a clade of contemporary viruses from The Netherlands (figure 2*a*,*c*). Although we cannot rule out that possibility that the H5N6 outbreak strain originated from an unsampled location, the spatio-temporal proximity and genetic similarity of the UK and The Netherlands strains means that it is plausible that the H5N6 outbreak was introduced to the UK by migrating birds from The Netherlands.

The single H5N6 virus that was sequenced from a mute swan on the Fleet Lagoon is placed as an outgroup to viruses sampled from a pochard duck (*Aythya ferina*) and a Canada goose (*Branta canadensis*) that were also HPAIV-positive at the Fleet Lagoon. The mute swan was known to be resident at the Fleet Lagoon. Given that infections in the mute swans were likely acquired locally, and the MRCA of the pochard and goose sequences is more recent than that of the swan, the pochard and goose may also have been infected locally.

### (c) Age distribution of mortality

A consistent pattern of age-structured mortality was observed in all three H5 HPAIV epidemics that occurred on the Fleet Lagoon: juvenile birds were more likely to die than any other age group during each of the H5N1, H5N8 and H5N6 outbreaks (figure 1*b*). The greatest mortality in juvenile birds occurred during the H5N8 outbreak, when 36% of juvenile birds died. We tested the significance of the difference in mortality between juvenile (less than 1 year) and older birds for each outbreak using two-sided Fisher's exact tests. Juvenile birds died 16.8 times more frequently than birds of all other ages during the H5N1 outbreak (95% confidence interval of odds ratio = 3.5–106.9; *p*-value less than 0.001). Juvenile birds died 10.2 times more frequently than birds of other ages during the H5N8 outbreak (95% confidence interval of odds ratio = 6.5–16.2; *p*-value less than 0.0001) and 71.0 times more frequently than birds of other ages during the H5N6 outbreak (95% confidence interval of odds ratio = 18.2–609.9; *p*-value less than 0.0001). By contrast, during the same winter period of previous non-epidemic years (2009–2015), juvenile birds were not significantly more likely to die than adult birds (odds ratio 1.6; 95% confidence interval of odds ratio = 0.9–3.1; *p*-value greater than 0.05) (electronic supplementary material, table S1).

### (d) Investigating the effect of age and weight on chance of death in juveniles

At the start of the H5N8 and H5N6 outbreaks (December 2016 and 2017), juvenile birds varied between 186 and 226 days old and 203 and 239 days old, respectively. Their last known weights, measured in September and October of their respective year of hatching, varied between 4.0 and 12.2 kg. Generalized linear models were constructed to test whether juvenile birds that died during the H5N8 and H5N6 outbreaks had, on average, different ages or weights from juvenile birds that survived. When analysing the outbreaks both together and separately, there was a trend towards older juveniles and those that were below average weight upon ringing being more likely to survive the outbreak, but neither difference was significant (*p*-value greater than 0.05) (electronic supplementary material, figure S3).

### (e) Serological responses

**Figure 3.**
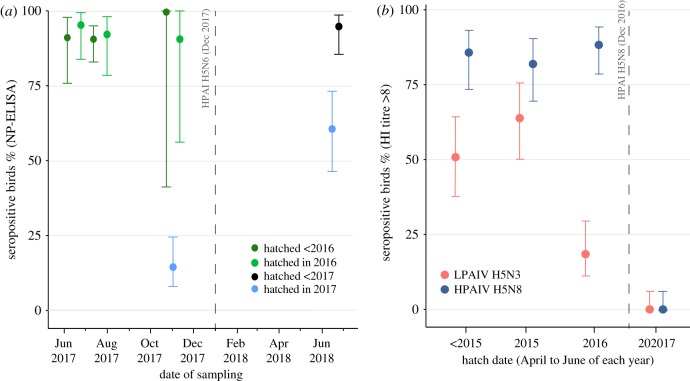
Seropositivity of swans. (*a*) Percentage of swans with antibodies targeting AIV NP by year of hatching and date of sampling. Colours represent birds hatched in different years. Adjusted Wald 95% confidence intervals are given. The date of the HPAI H5N6 outbreak is marked. (*b*) Percentage of swans with titres greater than 8 for HPAIV H5N8 (A/Turkey/England/16) (blue) and LPAIV H5N3 (A/Teal/England/06) (orange). Birds that were sampled on more than one occasion have been removed. If a bird was found to be seronegative for previous AIV exposure by NP-ELISA, it is included here as having a titre less than or equal to 8, despite HI assays not being conducted for that sample. Samples were collected during June, July and November 2017, but birds are grouped by hatch year only as no seasonal trend in change in titre was observed in these data.

In order to investigate why mortality was significantly higher in juveniles than in older birds during the three HPAIV outbreaks, we conducted serological assays on blood collected from swans in our study population at multiple times during 2017–2018. Throughout the year, the prevalence of antibody reactivity to AIV NP in swans greater than or equal to 2 years of age was greater than 90% (figure 3*a*, green and black). By contrast, juveniles that hatched in spring 2017 and which were tested at approximately five months of age in November 2017, had a prevalence of antibody reactivity of only 14% (figure 3*a*, blue). When this cohort (i.e. birds hatched in 2017) was tested again in early 2018, following the H5N6 outbreak, prevalence of antibody reactivity in juveniles had risen to 61% (figure 3*a*, blue).

HI assays were conducted on sera collected during June, July and November 2017 that tested positive for the presence of antibodies against AIV NP. Most birds that were alive at the time of the H5N8 outbreak (hatch year = 2016 or earlier) were seropositive for H5N8 (figure 3*b*). Among this group, birds that had hatched in spring/summer 2016 (and were therefore less than 1 year old when the H5N8 outbreak occurred in late 2016) were significantly less likely to be seropositive for LPAIV H5N3. None of the birds that hatched after the H5N8 outbreak (hatch year = 2017) exhibited any evidence of serological exposure to H5 AIV when tested in November 2017.

### (f) Association of serological responses with mortality during H5N6 2017/18

For the first time to our knowledge, we were able to explore whether the serostatus of wild birds tested prior to the H5N6 outbreak correlated with their likelihood of dying during that outbreak. Four of the 21 birds that died of H5N6 viral infection during winter 2017/18, and for which earlier blood samples were available, showed serological evidence of previous exposure to AIV by NP-ELISA. None of the 21 birds that died showed evidence of previous exposure to an H5 virus (titres less than 16), suggesting that these AIV seropositive birds had been exposed only to non-H5 viruses (electronic supplementary material, tables S2 and S3). While this does not exclude the possibility that previous exposure to *specific* non-H5 AIV might be protective against death from H5 HPAIV infection, it does suggest that previous exposure to H5, particularly to related strains, may be protective, and that not all LPAIVs are protective.

### (g) Seroconversion in individual birds and antibody duration

Blood was sampled from 59 swans on more than one occasion from June 2017 to June 2018. Of these, 11 birds seroconverted to be seropositive for antibodies targeting AIV NP (electronic supplementary material, figure S4). Only one bird showed evidence of the opposite trend (sero-reversion). Twelve birds were tested on two separate occasions (June/July and November 2017) for the presence of H5-specific antibodies using HI assays. Nine of the 12 birds had HI titres that remained stable or changed only twofold over this period. Only two of the 12 birds exhibited a reduction in titre of at least fourfold for HPAI H5N8 (electronic supplementary material, figure S5). Therefore, antibody responses to H5N8 HPAIV in many members of this population appear to be present for at least 11 months after primary infection.

### (h) Estimation of mortality rate among infected juvenile birds

A total of 64 live swans were swabbed during the peak of the H5N6 outbreak in 2017/18 (electronic supplementary material, figure S1). Of these, six had cloacal and/or oropharyngeal swabs that were positive for HPAI H5N6, with RT-qPCR cycle threshold (*C*_t_) values less than 37 (five hatched in 2017 and one hatched in 2014). In total, approximately 19% (5 out of 26) of live juvenile birds that were swabbed during the H5N6 outbreak tested positive for the virus, whereas only approximately 3% (1 out of 37) of live adult birds were positive. However, this difference was not statistically significant (Fisher's exact test; odds ratio = 8.3; *p* = 0.07). Two of the juvenile birds that were swabbed when alive and that tested positive for H5N6 during the outbreak subsequently died, at 3 and 11 days after swabbing. Both were confirmed to be positive for H5N6 HPAIV at death. A bird that hatched in 2014 and one of the birds that hatched in 2017 were sighted in early summer 2018, so both clearly survived infection.

Three times fewer birds died during the H5N6 outbreak than the H5N8 outbreak, and this reduction in mortality was observed in both juvenile and adult birds. Assuming that juvenile birds in each year were similarly immunologically naive, the reduction in deaths in juvenile birds could theoretically have occurred because fewer juveniles overall were infected, and/or because H5N6 was lower in virulence than H5N8. To determine whether H5N6 was less virulent in this population than H5N8, we estimated the case fatality ratio among juvenile birds for both outbreaks. Under several assumptions (detailed in Material and Methods), we estimate that mortality rates among *infected* juvenile birds may have been approximately 46% for HPAIV H5N8 and approximately 36% for HPAIV H5N6. While this might suggest a difference in HPAIV virulence in this population (and perhaps among related waterbirds), uncertainty in our case fatality estimates is high and, given the modelling assumptions made, we cannot rule out that the case fatality rates were the same in both outbreaks (electronic supplementary material, figure S2). The estimates of case fatality rate are consistent with the observation that between two and four of the five juvenile birds that tested positive for HPAIV during the H5N6 outbreak later died. It is therefore possible that the number of infected birds was simply lower during the H5N6 outbreak than the H5N8 outbreak—perhaps a result of partial herd immunity due to the previous exposure of the population to H5N8.

## 4. Discussion

Although the geographical and temporal spread of H5 HPAIV has been well characterized over large scales [5–8], detailed information about the dynamics and demographics of HPAI outbreaks in individual wild bird populations is rare. While longitudinal surveys of the epidemiology of LPAIV in wild birds are well established (e.g. [33,34]), the apparent unpredictability of HPAI outbreaks makes such studies more challenging for HPAIV. In this study, we present data from a wild population of long-lived birds that has experienced a series of outbreaks of H5 HPAIV, including the H5N8 and H5N6 epidemics presented here, and the H5N1 2007/08 event that has been reported in more detail previously [12]. We show that the timing, duration and drivers of mortality in these outbreaks are strikingly consistent between years, hinting that HPAIV may be more amenable for study in the wild than previously thought.

The estimated ‘start dates’ of all three outbreaks on the Fleet Lagoon are unexpectedly similar. This holds true both if the ‘start date’ is considered to be the first detection of positive birds, or if it is considered to be the date of the MRCA of the outbreak clade, obtained using phylogenetic reconstruction. The similarity in start dates among years is unexpected given the very complex ecology involved, and the absence of clear, repeatable trends at larger continental scales. Because many waterbird species cannot feed if wetland habitats freeze, wild bird movement and migration is known to be influenced by changes in local temperatures, which can in turn influence the geographical spread of AIV [35,36]. Autumn and early winter temperatures were higher in Europe in 2017 compared with 2016, and consequently, waterbirds generally arrived in the UK later and in lower numbers in 2016 than in 2017 [37]. Given the inter-annual variation in European climate and avian movement, it is therefore surprising that all three outbreaks at the Fleet Lagoon began at very similar times. However, data from the Fleet Lagoon suggest that the peak autumn counts of many different species occurred in the same month of all 3 years (electronic supplementary material, figure S6), so it is possible that bird immigration to the Fleet Lagoon was less variable between years than that at other locations. More detailed GPS tracking of individual migrating and resident birds at the Fleet Lagoon would help to resolve the effects of season and temperature on bird movement and density, which may explain the coincidental timing of the outbreaks observed here.

For H5N1, H5N8 and H5N6, the MRCA of all outbreak HA sequences was estimated to exist in mid- to late November of each year (95% HPD credible interval approximately October–December) [12], which makes it possible that each HPAIV circulated cryptically for up to ten weeks on the Fleet Lagoon before the first HPAIV-infected swans were detected. For several reasons, it seems unlikely that the virus could have circulated among the swans for as long as ten weeks before mortality manifested itself in the epidemic. Large numbers of swan faecal or cloacal samples collected at the site on 24 November 2016 (*n* = 69) and 3 November 2017 (*n* = 100) tested negative for AIV RNA by RT-qPCR. Given a population size of approximately 800 birds, we can be 95% confident that less than approximately 3.5% of the population could have been infected with HPAI H5 viruses on these dates. Experimental studies of H5N1 have demonstrated that most immunologically naive swans die of H5N1 infection between 5 and 10 days post-infection [38,39]. Here, we observe two birds that died 3 and 11 days after H5N6 HPAIV RNA was found in swabs taken from them. Further, there was an explosive increase in mortality following the first detected deaths in the H5N8 and H5N8 outbreaks (figure 1*a*). It therefore seems highly unlikely that the virus could have been circulating within the swan population for longer than a fortnight before increased mortality was observed. However, the virus could have been introduced by, and circulated undetected among, other species of waterbirds on the Fleet Lagoon, prior to the subsequent incursion into the swan population, as was previously suggested for H5N1 [12].

We cannot determine which species may have originally brought H5 HPAIV to the Fleet Lagoon. While the swan population suffered severe mortality that was consistent with the morbidity observed in experimental challenge studies [39,40], no significant increase in mortality was observed for other species. Two Canada geese and a common pochard were found dead and HPAIV-positive on the Fleet Lagoon (*Branta canadensis*, *n* = 1 during H5N6 and *n* = 1 during H5N1; *Aythya ferina, n* = 1 during H5N6). However, phylogenetic analysis cannot rule out that these birds were infected locally [12]. It is possible that the virus was introduced by a species that can tolerate HPAIV infection without showing disease symptoms [41]. We inferred that all H5 HPAIVs entered the Fleet Lagoon at the time of year when waterbird immigration to the Fleet Lagoon is highest, as previously noted for H5N1 [12]. Species that migrate into the Fleet Lagoon during autumn and early winter (such as the common pochard and other long-distance migratory species) are therefore more plausible vectors of the virus than those species that are locally resident (such as the mute swan or Canada goose) (electronic supplementary material, figure S7), supporting the results of phylodynamic analyses conducted at an international scale [4]. Analysing detailed bird count data from affected sites in different countries, with better spatio-temporal tracking of bird movements, may help to determine which species drive the long-distance movement of HPAIV.

Using daily counts of swans that were found dead at the site, we estimated the mean basic reproductive number (*R*_0_) for lethal infection as approximately 2.5 for both outbreaks. This is slightly higher than *R*_0_ values estimated for H5N1 from case count data (mean *R*_0_, 1.6 [42]) and for clade 2.3.4.4 viruses H5N2 and H5N8 via phylodynamic modelling (mean *R*_0_ from HA gene: 1.6–1.7 [43]). This may be because the Fleet Lagoon swan population comprises a high proportion of immunologically naive, juvenile birds among which the virus could be easily transmitted, or because of the high bird density at the site that is encouraged by regular, supplementary feeding.

For the first time, we report data on mortality outcomes of individual wild birds, whose serological status had been observed immediately prior to natural exposure to HPAIV. Although several birds that died of HPAIV were seropositive for previous AIV infection two months prior to the HPAIV outbreak, none of these birds had been previously infected with an H5 virus. This finding is not appropriate for statistical testing owing to the low sample sizes. However, it is certainly consistent with mounting evidence from experimental challenge studies, and from observational data on LPAIV, that birds are more protected against infection by a novel AIV if they were previously infected by a virus of the same subtype than by a virus of a different subtype [11,44–48].

Whether age correlates with protection against HPAIV is difficult to study in wild bird populations, as population age structures are rarely known and HPAIV infection is typically rare. Previous exposure to certain LPAIVs can reduce morbidity upon challenge with H5 HPAIV [11,40,44,49]. As birds age, they are increasingly likely to gain serological responses to AIV, including to an increasingly broad range of different subtypes [50–60]. This immunological pattern could lead to older birds having a lower risk of death upon HPAIV infection. Among swans on the Fleet Lagoon, we found that birds that were greater than 1 year old at the time of infection with HPAIV were significantly less likely to die than younger birds during all three outbreaks. In addition, testing of live birds during the peak of the H5N6 outbreak in 2017/18 showed that older birds were eight times less likely to have the qPCR-detectable virus than younger birds, although this difference was not significant. Swan age was also closely correlated with immunological responses against AIV NP and also against H5 HA. Despite suggestions that slight differences in age (weeks to months) may modulate mortality risk upon HPAIV exposure, even in the absence of differences in immunological status [61,62], we found no evidence of this among birds on the Fleet Lagoon and our data indicate that immunological status is the key driver of mortality risk in this population.

We found no evidence for seasonal differences in AIV seroprevalence in the swans on the Fleet Lagoon, in agreement with our previous conclusion that antibodies to AIV are likely to be long lasting in swans [63]. Immunologically naive birds are therefore mainly introduced into the population only via hatching and not via sero-reversion. It is possible that age-related patterns of mortality are less clear in other avian species, for example, if adult birds sero-revert more frequently, rendering less distinct the immunological profiles of adults and juveniles [55,64]. The prevalence of immunological responses to a virus in a population is a critical determinant of whether the virus can be maintained in the absence of antigenic adaptation. The rate of introduction of immunologically naive birds into bird populations, mediated by differences in lifespan and the duration of antibody responses, should be investigated in more detail for a wider number of species in order to better understand the risk of H5 HPAIV becoming enzootic in wild birds.

Decades of research into human pathogens has proven the importance of cohort studies and individual-level demographic data for understanding infectious disease epidemiology. Despite this, individual-level data are almost never available for outbreaks in wild animals or plants, and the unit of study is instead often a larger group (e.g. flock, herd or farm). Individual-level studies that generate high-quality data are often impractical or too expensive to implement in the wild. Here, we were able to exploit bird demographic data that were collected for the purpose of longitudinal ecological research, in order to investigate the demographic context of virus transmission in wild animals. This approach has potential applications to other emerging outbreaks and could be facilitated by close collaboration between ecologists, virologists and epidemiologists at high-risk sites. Such analyses have the potential to generate epidemiological information for wild animal and plant populations that is of the same quality as that achievable by prospective cohort studies of human populations.

## Supplementary Material

Supplementary Material

## Supplementary Material

Supporting Data

## Supplementary Material

Maximum clade credibility tree for HA segment

## Supplementary Material

BEAST XML file for HA segment phylogeny

## Data accessibility

Sequences generated here are available from GenBank (H5N8 sequences, accession numbers MH819084-MH819175) or GISAID (H5N6 sequences, GISAID identifiers EPI ISL 292223–292225). The primary data used to generate the results presented here, including BEAST XML and MCC tree files, and acknowledgements of the laboratories contributing sequences to GISAID that were used in phylogenies, are available in the electronic supplementary material.
