# Supplementary material for: Comparative micro-epidemiology of pathogenic avian influenza virus outbreaks in a wild bird population
Source: Philos Trans R Soc Lond B Biol Sci. 2019 May 6;374(1775):20180259. doi: 10.1098/rstb.2018.0259 (PMC6553603; doi:10.1098/rstb.2018.0259)
Supplement: Supplementary Material [file rstb20180259supp1.docx]

**Supplementary Material: Comparative micro-epidemiology of pathogenic avian influenza virus outbreaks in a wild bird population**

Sarah C Hill ^1^*, Rowena Hansen ^2^, Samantha Watson ^2^, Vivien Coward ^2^, Christine Russell ^2^, Jayne Cooper ^2^, Steve Essen ^2^, Holly Everest ^2^, Kris V Parag ^1^, Steven Fiddaman ^1^, Scott Reid ^2^, Nicola Lewis ^2,4^, Sharon Brookes ^2^, Adrian L Smith ^1^, Ben Sheldon ^1,3^, Christopher M Perrins ^1,3^, Ian H Brown ^2^, Oliver G Pybus ^1^*

1. Department of Zoology, University of Oxford, United Kingdom
2. Department of Virology, Animal and Plant Health Agency - Weybridge, Woodham Lane, New Haw, Addlestone, Surrey, KT15 3NB, United Kingdom
3. Edward Grey Institute, Department of Zoology, University of Oxford, United Kingdom
4. The Royal Veterinary College, Royal College Street, London, UK.

**Supplementary Table 1: Number of birds that died during the ‘outbreak period’ of 23^rd^ December to 24^th^ January of each alternate year between 2009-2015, and sum of the estimated population sizes for those years.**

| **Period (Dec-Jan)** | **Estimated count of birds that died** | | **Remaining birds** | |
| --- | --- | --- | --- | --- |
|  | **Hatch year** | **Other ages** | **Hatch year** | **Other ages** |
| **2009-2015** | 17 | 39 | 970 | 3753 |

**Supplementary Table 2: Observed deaths and presumed survival of birds tested by ELISA for presence of antibodies targeted at AIV NP.**

|  | **NP AIV seropositive^1^ (ELISA)** | **NP AIV seronegative (ELISA)** |
| --- | --- | --- |
| **Died and H5 positive** | Juveniles: 3  Adults: 1 | Juveniles: 17  Adults: 0 |
| **Presumed survived^2^** | Juveniles: 7  Adults: 187 | Juveniles: 43  Adults: 16 |

^1^ Note that NP AIV seropositive birds may or may not also be seropositive for H5 AIV.

^2^These birds were not observed to die during or prior to the H5N6 AIV outbreak so are assumed to have survived.

**Supplementary Table 3: Observed deaths and presumed survival of birds tested by HI assays for presence of antibodies targeting AIV H5 HA protein**

|  | **H5 AIV seropositive (HI assay)** | **H5 AIV seronegative (HI assay) or NP AIV seronegative (ELISA)^2^** |
| --- | --- | --- |
| **Died and H5 positive** | Juveniles: 0  Adults: 0 | Juveniles: 20  Adults: 1 |
| **Presumed survived ^1^** | Juveniles: 0  Adults: 147 | Juveniles: 50  Adults: 25 |

^1^These birds were not observed to die during or prior to the H5N6 AIV outbreak so are assumed to have survived.

^2^5 birds testing negative for NP were tested by HI assay to confirm H5 negative. All 5 birds had titres <8, so other birds that are NP negative are therefore assumed here to be also H5 seronegative.

**Supplementary Figure 1:**

Mortality observed in the population during outbreaks of A) H5N8 (Dec 16/Jan 17) and B) H5N6 (Dec 17 / Jan 18). Grey bars indicate birds that were not tested by RT-qPCR for AIV. Birds that died during the period indicated with an asterix are all assumed to be HPAIV positive but other untested birds are not. The black arrow indicates the date of live bird testing for AIV, which coincided with the peak of mortality during Jan 18.

**
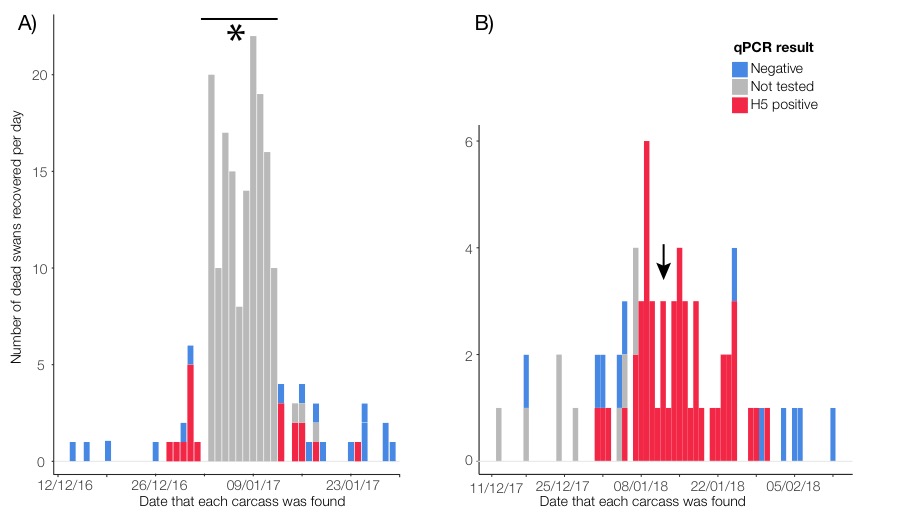
**

**Supplementary Figure 2:**

Mortality rate distributions for H5N8 (blue) and H5N6 (red). For a total swan population (N) drawn uniformly from [N_min_ N_max_], a posterior distribution was computed for the proportion of birds (p = number immune (x)/number sampled (n)) that have been infected but recovered, (R), for each outbreak. A conjugate Beta(0.5, 0.5) Jeffrey’s prior, which is uninformative, was used to derive the posterior on p, which is Beta(0.5+x, 0.5 + n-x) under a Binomial(n, p) model. This yields a posterior on R as p(N-D). A point estimate of D was then used to calculate the distribution of the mortality rate.


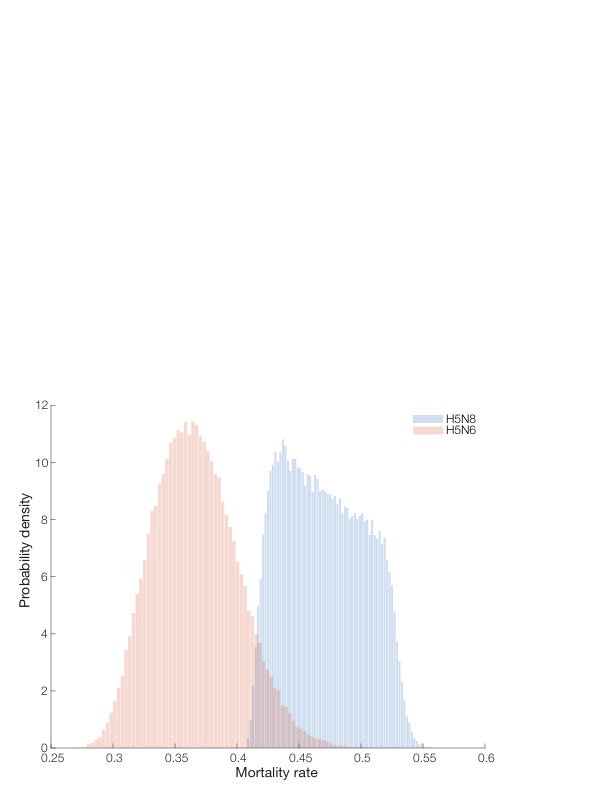
N_max_ is the estimated numbers of birds in the population (see Materials and Methods) and represents a likely maximum population estimate, as birds that have died unobserved since the last accurate census would be included in its value. N_min_ was calculated for each outbreak, based on the number of birds observed at the next occurring census following each outbreak and including any birds observed between the outbreak and the census. Because an accurate census has not been taken since H5N6 2017/2018, N_min_ for H5N6 was calculated as 80% of N_max_ for H5N6, following the differences between H5N8 N_max_ and N_min_ population estimates.

**Supplementary Figure 3:**


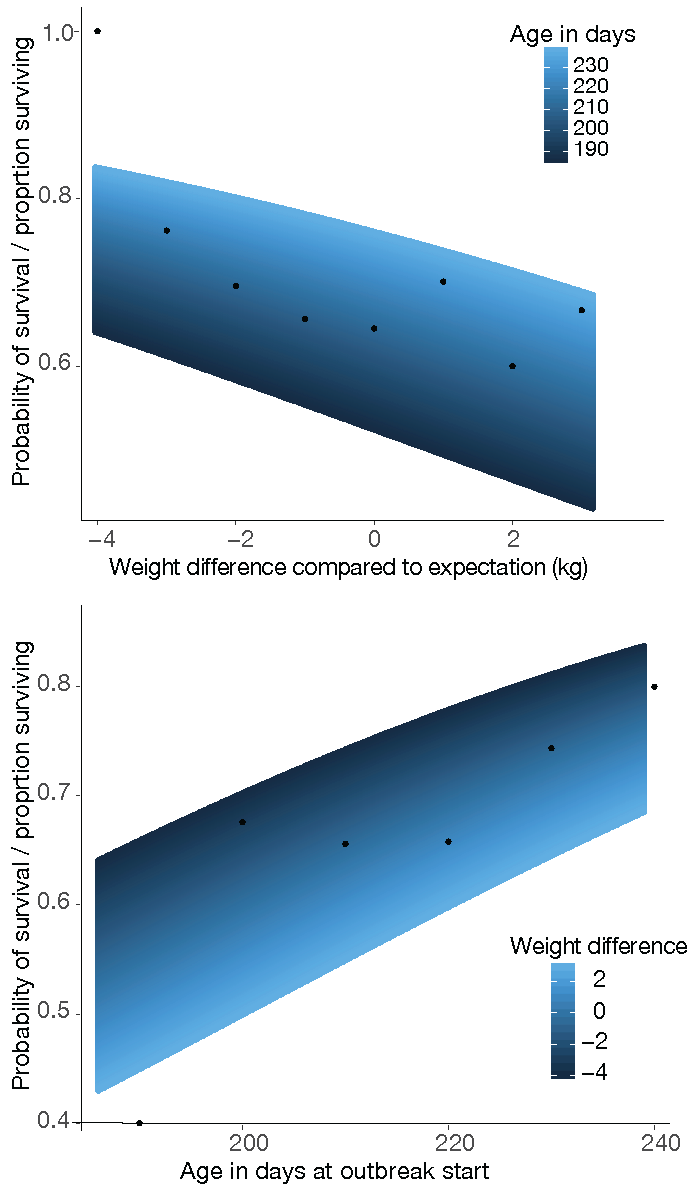
Fitted probability of a juvenile bird surviving H5N8 and H5N6 given ‘weight difference’ at ringing (upper) and age at the start of the outbreak (lower), calculated using GLMs. ‘Weight difference’ is the difference in weight for a juvenile compared to that expected for a bird of its exact age and sex on the day it was ringed (values >0 correspond to birds that were heavier than expected). Coloured bars represent the fitted probability of survival, and black dots represent the proportion of birds that survived from each binned age or weight group, with bins of size 10 days and 1 kg, respectively.

**Supplementary Figure 4:**

Serostatus of birds sampled >1 time during 2017-2018 by NP ELISA. Colours indicate change in serostatus between each sample. Age of bird is indicated when known.

**Supplementary Figure 5:**

Change in HI titre between different samples for birds tested by HI assay in June/July 2017 and in November 2017. Dots indicate titre in June/July, and arrows indicate directionality of change and new titre in November 2017, if a change occurred. Colours indicate different antigens.

**Supplementary Figure 6:**

Average seasonal counts of birds on the Fleet Lagoon of the three species in which HPAIV H5N6 was found. Bird counts are rescaled such that the highest median count is equal to 1 for all species. Width of the line indicates the number of birds present on the site during that month.

**Extended Materials and Methods**

*Blood sampling*

Blood was drawn from either the brachial vein or superficial leg vein and transferred to Starstedt 1.1ml Z-Gel tubes. Following clotting, serum was separated by centrifugation at 1000 g for 10 minutes and transferred to new tubes.

*Treatment of sera prior to serological assays*

Sera were incubated at 56 C for 30 minutes to destroy complement prior to serological assays. Sera were also treated with receptor-destroying enzyme and red blood cell adsorption prior to testing using haemagglutination inhibition (HI) assays. Specifically, one volume of heat-treated sera was added to four volumes of receptor-destroying enzyme. The mixture was incubated for 16 hours at 37C before heat-inactivation at 56C for 1 hour. Sera were cooled to 4C, and one volume of packed red blood cells from specific-pathogen-free chickens was added to five volumes of serum. The samples were incubated at room temperature for 30 minutes, centrifuged at 1000 g to pellet the red blood, and the separated serum was transferred to a clean tube. Sera were stored in 55 µl aliquots to reduce freeze-thawing during HI assays.

*HI assays*

HI assays were conducted according to standard methods (1). Before every HI assay, haemagglutination assays were conducted to determine 1 haemagglutination unit (HAU) of antigen from starting dilutions of 1/2, 1/3 1/5, 1/7 and 1/9 and the chosen 4HAU antigen dilution was checked by back-titration. A two-fold dilution series of sera was used in the HI assays from a starting dilution of 1/8. Cell control wells were run for every sample in both assays to check for the absence of non-specific haemagglutination, and positive antisera and negative control sera (taken from specific pathogen free chickens) were also tested for each assay.

*Viral RNA detection*

All samples were tested for the presence of AIV at the APHA. Individual oropharyngeal or cloacal swabs were eluted into 1ml brain heart infusion broth (BHIB) containing antibiotics (1000 IU penicillin G, 10 µg/ml amphotericin B, 1 mg/ml gentamycin) and 140 µl of swab-suspension was carried forward into nucleic acid extraction. RNA extractions were conducted using the QIAmp viral RNA BioRobot kit customised for the APHA in conjunction with a Universal BioRobot (Qiagen) as described previously (2). Samples were tested for the presence of AIV matrix gene RNA using a previously published protocol (3). Samples that were positive by this PCR assay were subjected to further PCR testing of H5 and H7 (2,4) haemagglutinin types, and H5 positive samples were subsequently tested for N8 and N6 neuraminidase types by additional PCRs (James et al., manuscript submitted for publication, 2018).

*Sequencing methods*

H5N8-positive samples from January 2017 were subjected to cDNA synthesis using the Protoscript First Strand cDNA Synthesis Kit (NEB) according to the protocols detailed in (5). A multiplex primer scheme was designed to amplify the whole viral genome of the strain. The scheme consisted of 92 different primers designed to amplify overlapping amplicons of 400bp each, with an overlap length of 75bp between neighbouring amplicons. PCRs were conducted according to previously published methods using 40 cycles of denaturation, annealing and extension . Amplified DNA and appropriate negative controls were sequenced in barcoded multiplexes of 6 - 8 samples per run on the MinION (Oxford Nanopore Technologies) using FLO-MIN106 flow cells and MinKnow 1.7.7. Library preparation and consensus sequence generation was each barcoded sample was conducted according to (5) (library reagents kits numbers; SQK-LSK108, EXP-NBD103). The reference genome used for mapping and Nanopolish variant calling was (A/turkey/England/052131/2016; GISAID Isolate ID EPI\_ISL\_239801).

H5N6-positives samples were grown in hen’s eggs and the RNA extracted using a Qiagen QIAamp Viral RNA Mini kit. Double stranded cDNA was generated using Roche cDNA Synthesis System according to the manufacturer’s instructions. 1ng of cDNA was used to prepare a sequencing library using Illumina NexteraXT reagents. The dsDNA was sequenced on an Illumina MiSeq. Data was processed using a publically available script (<https://github.com/ellisrichardj/FluSeqID>) (details in **Supplementary Material)**. Using this script, all reads were first mapped against a relevant host genome (*Gallus gallus*, NCBI assembly ID GCA_000002315.5_GRCg6a) using BWA (6). All unmapped reads were extracted using samtools (7) and assembled de-novo using Velvet (8). The resulting contigs were then used as queries in a BLAST search to generate a candidate influenza virus reference genome. The sequencing reads that did not map to the host were mapped to this reference genome, and a consensus genome was produced from these data using a second publically available script (https://github.com/ellisrichardj/csu_scripts). The mapping process was run iteratively 4 times to produce the best possible consensus genome, and the final assembled genome was checked and edited using Tablet (9).

**References**

1. OIE. Chapter 2.3.4, Avian influenza. In: Manual of Diagnostic Tests and Vaccines for Terrestrial Animals [Internet]. 2015 [cited 2016 Feb 1]. Available from: http://www.oie.int/fileadmin/Home/eng/Health_standards/tahm/2.03.04_AI.pdf

2. Slomka MJ, Pavlidis T, Coward VJ, Voermans J, Koch G, Hanna A, et al. Validated RealTime reverse transcriptase PCR methods for the diagnosis and pathotyping of Eurasian H7 avian influenza viruses. Influenza Other Respir Viruses. 2009 Jul;3(4):151–64.

3. Nagy A, Vostinakova V, Pirchanova Z, Cernikova L, Dirbakova Z, Mojzis M, et al. Development and evaluation of a one-step real-time RT-PCR assay for universal detection of influenza A viruses from avian and mammal species. Arch Virol. 2010 May;155(5):665–73.

4. Slomka MJ, Pavlidis T, Banks J, Shell W, McNally A, Essen S, et al. Validated H5 Eurasian real-time reverse transcriptase-polymerase chain reaction and its application in H5N1 outbreaks in 2005-2006. Avian Dis. 2007 Mar;51(1 Suppl):373–7.

5. Quick J, Grubaugh ND, Pullan ST, Claro IM, Smith AD, Gangavarapu K, et al. Multiplex PCR method for MinION and Illumina sequencing of Zika and other virus genomes directly from clinical samples. Nat Protoc. 2017 Jun;12(6):1261–76.

6. Li H. Aligning sequence reads, clone sequences and assembly contigs with BWA-MEM. ArXiv13033997 Q-Bio [Internet]. 2013 Mar 16; Available from: http://arxiv.org/abs/1303.3997

7. Li H, Handsaker B, Wysoker A, Fennell T, Ruan J, Homer N, et al. The Sequence Alignment/Map format and SAMtools. Bioinformatics. 2009 Aug 15;25(16):2078–9.

8. Zerbino DR, Birney E. Velvet: Algorithms for de novo short read assembly using de Bruijn graphs. Genome Res. 2008 May;18(5):821–9.

9. Milne I, Bayer M, Cardle L, Shaw P, Stephen G, Wright F, et al. Tablet—next generation sequence assembly visualization. Bioinformatics. 2010 Feb 1;26(3):401–2.
